# The Sea Lamprey as an Etiological Model for Biliary Atresia

**DOI:** 10.1155/2015/832943

**Published:** 2015-05-26

**Authors:** Yu-Wen Chung-Davidson, Chu-Yin Yeh, Weiming Li

**Affiliations:** ^1^Department of Fisheries and Wildlife, Michigan State University, East Lansing, MI 48824, USA; ^2^Department of Physiology, Michigan State University, East Lansing, MI 48824, USA; ^3^College of Osteopathic Medicine, Michigan State University, East Lansing, MI 48824, USA

## Abstract

Biliary atresia (BA) is a progressive, inflammatory, and fibrosclerosing cholangiopathy in infants that results in obstruction of both extrahepatic and intrahepatic bile ducts. It is the most common cause for pediatric liver transplantation. In contrast, the sea lamprey undergoes developmental BA with transient cholestasis and fibrosis during metamorphosis, but emerges as a fecund adult with steatohepatitis and fibrosis in the liver. In this paper, we present new histological evidence and compare the sea lamprey to existing animal models to highlight the advantages and possible limitations of using the sea lamprey to study the etiology and compensatory mechanisms of BA and other liver diseases. Understanding the signaling factors and genetic networks underlying lamprey BA can provide insights into BA etiology and possible targets to prevent biliary degeneration and to clear fibrosis. In addition, information from lamprey BA can be used to develop adjunct treatments for patients awaiting or receiving surgical treatments. Furthermore, the cholestatic adult lamprey has unique adaptive mechanisms that can be used to explore potential treatments for cholestasis and nonalcoholic steatohepatitis (NASH).

## 1. Introduction

Biliary atresia (BA) is a progressive, inflammatory, and fibrosclerosing cholangiopathy of infants that results in obstruction of both extrahepatic and intrahepatic bile ducts [1, 2]. Human BA is traditionally categorized into two forms: an embryonic/fetal form (approximately 20% of the cases) associated with other congenital anomalies and a perinatal/acquired form (80% of the cases) with postnatal injury followed by fibroobliteration of the bile ducts. However, there are many clinical variations that may be better classified into three groups: BA with other congenital malformations, cystic BA, and isolated BA [3]. Isolated BA is the most common form with wide geographical variation in incidence across the world from 1 in 5,000 births in Taiwan to 1 in 18,000 births in Europe [4–8]. This disease is more common in females, premature babies, and children of Asian or African American heritage [8]. Infants with isolated BA show symptoms of jaundice between 1 and 3 months of age. The Kasai portoenterostomy is often used to restore bile flow [9]. Timely performance of the Kasai operation has cured 20% of patients [4, 10–12]. However, despite medical and surgical intervention, the majority (80%) of patients develop progressive bile duct injury and fibrosis within the liver, ultimately leading to biliary cirrhosis [13] and liver failure [14]. These patients eventually require liver transplantation, accounting for half of pediatric liver transplants [15]. Nonetheless, the combination of Kasai procedure and liver transplantation is effective and provides a 10-year survival rate close to 90% [16–18].

Despite intensive clinical and basic research, it is still unclear what triggers BA and how to stop the ongoing liver deterioration [19, 20]. A clear solution is to discover the cause of BA. Unfortunately, most patients are diagnosed when the extrahepatic bile duct is already atretic and the liver has reached advanced fibrosis. Therefore, it is difficult to determine the etiological sequences and whether the accompanying inflammation is a primary or secondary phenomenon. Present etiological research in humans is restricted to the time of the Kasai procedure, which does not allow tracing BA pathology back to its origin. Many studies have examined morphological and immunological findings at the time of and after the Kasai operation, but all failed to explain the initial events [17].

In the mid-1960s, the quest for a simulation or animal model for BA began. Since then, many animal models have been proposed, but these studies have not led to clinical applications [17]. There are sporadic reports in the veterinary literature of BA or BA-like outbreaks in lambs, foals, dogs, and calves [21–23]. However, these reports are rare and have generated little interest and few follow-up studies [17]. Youson described naturally occurring developmental BA in lampreys [24], the only animal model in which the entire BA process can be traced from the beginning to the end. We will show new histological evidence and review recent findings to discuss how this animal model is well suited to answer questions for BA etiology.

## 2. The Progression of Developmental BA in the Sea Lamprey

A critical issue for utilizing the sea lamprey as a model for BA is whether it displays symptoms exemplifying human BA (Table 1). Earlier literature touched upon this issue but did not follow the progression of these symptoms to the extent that may provide insights of the etiology or possible adaptive mechanisms for recovery. Therefore, it is critical to investigate the progression of developmental BA to identify strategies for clinical treatment.

The sea lamprey, a jawless vertebrate, goes through several life cycle stages. After hatching, they exist as filter-feeding larvae in the bed of freshwater streams. In the juvenile phase, sea lampreys metamorphose into blood-feeding parasites in the Great Lakes or the ocean. They later become nontrophic and migrate to freshwater streams to spawn and die [25–27]. During metamorphosis from larvae to parasitic juveniles [28], the lamprey liver loses the entire biliary system, a process reminiscent of human BA [24, 29]. The mammalian and lamprey liver share similar histological and ultrastructural features [24, 30, 31]. Sea lamprey metamorphosis is a highly synchronized process, which in North America commences in early July and is complete in early November [24]. During this period, the exterior appearance transforms through seven stages [28]. In the liver, the basement membrane shows histological changes at metamorphic stages 1 and 2 and becomes dramatically reorganized at stages 3 and 4. Bile duct degeneration is asynchronous during sea lamprey metamorphosis: the extrahepatic bile ducts and the gall bladder are fully degenerated by metamorphic stage 3 while one or two intrahepatic bile ducts might persist into stages 5 and 6 but usually disappear by stage 7 [29].

The cholangiocytes lining the biliary tract degenerated via programmed cell death or apoptosis [32, 33]. Morii et al. used terminal deoxynucleotidyl transferase dUTP nick-end labeling (TUNEL) and caspase-3 immunohistochemical staining to analyze the spatial and temporal sequences of cholangiocyte apoptosis during metamorphosis in Japanese lamprey [33]. At the onset of metamorphosis (late larval stage), nuclear staining of active caspase-3 (apoptotic marker) was detected in the cystic duct (CD) and large intrahepatic bile ducts (IHBDs), and cytoplasmic staining of active caspase-3 was observed in medium IHBDs [33]. At early metamorphic stage, nuclear staining of active caspase-3 was shifted to medium IHBDs and extrahepatic bile duct (EHBD), and cytoplasmic staining of active caspase-3 was observed more peripherally in small IHBDs [33]. The gall bladder, CD, and large IHBDs all showed DNA fragmentation at this stage [33]. The bile canaliculi between hepatocytes were dilated and displayed features similar to cholestasis [33]. At late metamorphic stage, the entire biliary system degenerated [33], consistent with the findings in sea lamprey [24, 29, 32].

**Figure 1:**
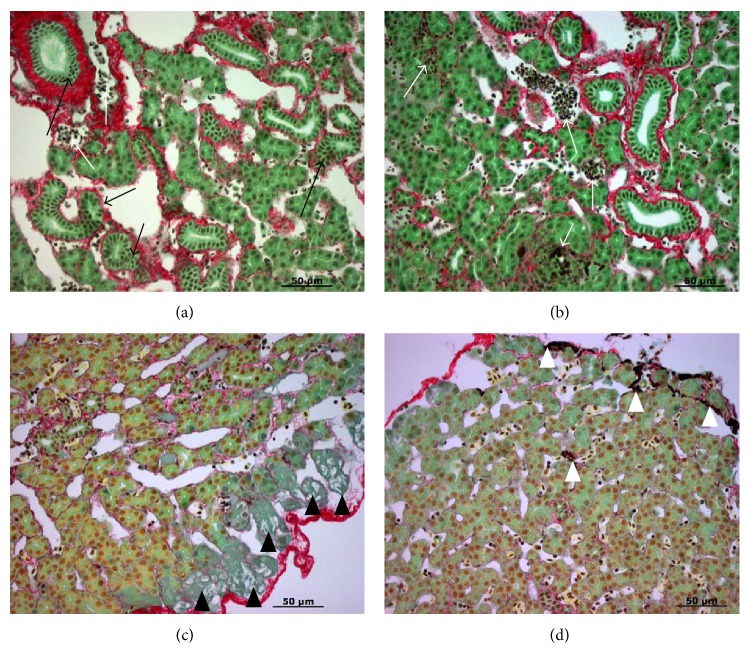
Liver morphology at the onset of metamorphosis. (a) Cholangiocyte proliferation (black arrows) and lymphocyte infiltration (white arrows). Collagen staining (red) is located at the basement membrane and slight thickened around proliferating bile ducts. (b) Lymphocyte infiltration (white arrows) at hepatocyte foci and extracellular spaces. (c) Hepatocyte necrosis and autolysis (solid black triangles) around the edge of the liver. (d) Dead cells and fibrotic debris (solid white triangles). Paraffin sections were stained with Picro Sirius Red (Gladstone). Scale bar: 50 *μ*m.

**Figure 2:**
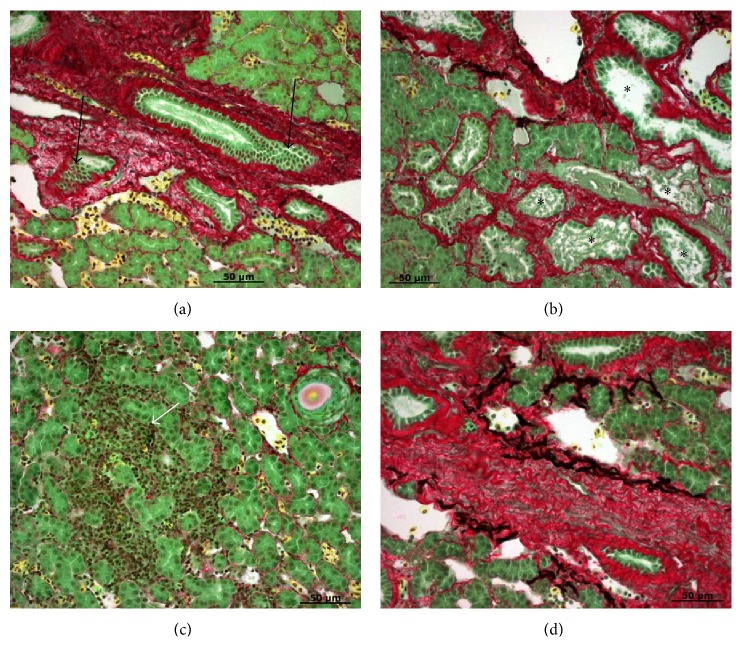
Liver morphology at metamorphic stages 1 ((a) and (b)) and 2 ((c) and (d)). (a) Heavy fibrosis (red stain) around proliferating bile ducts (black arrows) and activated macrophages (yellow cells with filopodia) clearing the fibrosis and dead cell debris. (b) Cholangiocyte proliferation and autolysis (asterisks). (c) Lymphocyte infiltration at hepatocyte loci (white arrow). (d) Heavy fibrosis (red stain) containing dead cells and fibrotic debris (black). Paraffin sections were stained with Picro Sirius Red (Gladstone). Scale bar: 50 *μ*m.

**Figure 3:**
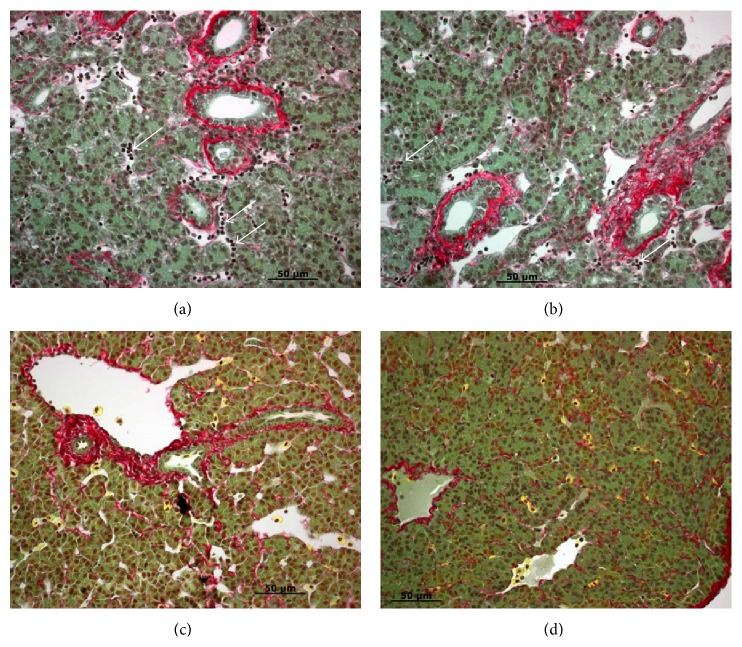
Liver morphology at metamorphic stage 3 ((a) and (b)) and in newly transformed juveniles ((c) and (d)). ((a) and (b)) Lymphocyte infiltration (white arrows) at extracellular spaces. Fibrosis (red stain) is reduced and activated macrophages (yellow cells with filopodia) are no longer present. ((c) and (d)) Collagen fibers (red stain) at the basement membrane and activated macrophages (yellow cells with filopodia) clearing the residual dead cells and fibrotic debris (black). Paraffin sections were stained with Picro Sirius Red (Gladstone). Scale bar: 50 *μ*m.

Inflammation and fibrosis during developmental BA were mentioned but not very distinct in early literature [34, 35]. In a recent study, we used Picro Sirius Red collagen staining to examine liver fibrosis at the onset of metamorphosis (Figure 1), metamorphic stages 1 (Figures 2(a) and 2(b)), 2 (Figures 2(c) and 2(d)), and 3 (Figures 3(a) and 3(b)), and newly transformed juvenile (Figures 3(c) and 3(d)). At the onset of metamorphosis, lymphocyte infiltration was observed in hepatocyte foci and extracellular spaces (Figures 1(a) and 1(b)). Cholangiocyte proliferation, pericholangitis, and thickening of the basement membrane were also obvious (Figure 1). Some hepatocytes at the outer edge of the liver started to show signs of necrosis and autolysis (Figure 1(c)). At metamorphic stage 1, cholangiocytes continued to proliferate and autolyze into the lumen (Figures 2(a) and 2(b)), but lymphocyte infiltration was only observed in the extracellular spaces. The number of activated macrophages (yellow cells with filopodia) increased dramatically at this stage (Figures 2(a) and 2(b)). Heavy fibrosis was observed around bile ducts, with macrophages clearing the fibrotic and dead cell debris (Figures 2(a) and 2(b)). At metamorphic stage 2 (Figures 2(c) and 2(d)), a second bout of lymphocyte infiltration was observed at hepatocyte foci (Figure 2(c)). Cholangiocytes continued to proliferate and autolyze into the lumen. Heavy fibrosis persisted and activated macrophages continued to clear the fibrotic and dead cell debris (Figure 2(d)). At metamorphic stage 3, activated macrophages seemed to recede and the degree of fibrosis was reduced, but lymphocytes were still abundant at the extracellular spaces (Figures 3(a) and 3(b)). In newly transformed parasitic juvenile, most collagen was deposited around the blood vessels at the basement membrane, and a few activated macrophages continued to clear the residual fibrotic and dead cell debris (Figures 3(c) and 3(d)). These results are consistent with earlier observations of lymphocyte infiltrations at hepatocyte foci and the presence of Kupffer cells (residential macrophages) during metamorphosis [34]. The ultrastructure of the macrophages often contained electron-dense granules and degenerating cells in large intracellular vacuoles. In addition, collagen microfibrils and some microfilaments were observed in the extracellular spaces [36]. These results are also consistent with histological features of human BA such as bile duct obliteration and proliferation, lymphocyte infiltration of the periportal areas, and progressive bridging liver fibrosis [37].

Human BA studies have documented the influx of macrophages into portal tracts [38, 39]. The relationship of macrophages with bile duct injury has also been shown in other human biliary diseases. Cameron et al. reported persistent activation of macrophages in livers with primary sclerosing cholangitis, which contributed to chronic release of TGF*γ*, inflammation, and fibrosis [40]. Based on the progression of lamprey developmental BA, it is possible to identify the factor(s) associated with the first signs of inflammation at the onset of liver metamorphosis that may be triggered by metamorphic signal(s). These results can then be confirmed in other animal models or be utilized to search patient history for similar exposure. It is also possible to identify the factor(s) that activate(s) the macrophages to clear rather than to induce chronic inflammation and fibrosis. Hepatic fibrosis leading to cirrhosis and eventually liver transplantation is the major morbidity in BA patients [41, 42]. Liver fibrosis is also the common injury response regardless of the type of hepatic injury or the source of fibrogenic cells. Therefore, one of the therapeutic targets for liver injury is to suppress the progressive fibrosis [43]. If the fibrosis clearing factors were identified, many liver diseases could potentially be remedied. The lamprey model could provide the signaling mechanism to clear fibrosis, which would be useful for clinical management of patients with advanced fibrosis.

## 3. Comparisons of Existing Animal Models with Lamprey Model

To illustrate the advantages and possible limitations of the sea lamprey model, we compare it with the existing animal models. In this section, we will highlight the existing models first and then comment on the lamprey model.

Although the etiology of BA is unclear, there are several theories of pathogenesis derived from the existing animal models, including viral infection [2, 44–46], autoimmune-mediated bile duct destruction [47, 48], and abnormalities in bile duct development [49]. Current opinion is that it may involve a primary perinatal hepatobiliary virus infection and a secondary inflammatory or autoimmune-mediated bile duct injury [50]. This view can explain some but not all cases of BA. Numerous attempts have been made to isolate hepatotropic viruses from the liver of children with extrahepatic biliary atresia (EHBA). At different stages of the disease, human papillomavirus [51], cytomegalovirus [52], respiratory syncytial virus [53], and rotavirus and reovirus type 3 [49, 54–56] were identified. However, none of them are specifically correlated with BA [57].

### 3.1. Bile Duct Ablation Models

Drug administration during pregnancy can induce experimental BA in some animal models [58, 59]. A number of postnatal maneuvers (intrabiliary injection of sclerosants and superglue) in a variety of animal models can evoke early neonatal cholestasis [60–67]. These models replicate the metabolic consequences of cholestasis and the pathogenesis of cholangitis but do not provide real insights into BA etiology [17].

Injection of carbon tetrachloride (CCl_4_) can induce hepatic fibrosis [43] and necrosis of hepatocytes around the central vein and connective tissue septa linking portal canals and central veins [66, 67]. Prolonged biliary obstruction with CCl_4_ for 15 days or more can lead to cirrhosis [68–73]. However, there are two major obstacles to produce consistent and predictable cirrhosis with this model. First, the individual response to CCl_4_ is variable. Second, mortality during the first week of treatment is 20–60% and varies widely depending on the animal strains [74]. In addition, this method takes 10–12 weeks to produce cirrhosis [71], and CCl_4_ intoxication is extremely rare in humans [74, 75].

A rat model of liver cirrhosis induced by total intrahepatic biliary ablation with pure ethanol has been reported [76]. In this model, the liver showed higher levels of total bilirubin, aspartate aminotransferase, alanine transaminase, and hyaluronic acid after 8 weeks of treatment. The expression of *α*-smooth muscle actin, a marker for myofibroblasts, was prominent in the surrounding proliferating bile ducts and portal areas. The distribution of TGF*β*1, a fibrogenic factor, was found predominantly in hepatocytes in the center of nodules and in ductular epithelial cells [76]. 24% of the ethanol-injected animals survived to 8 weeks, and some of them developed progressive sclerosing cholangitis and intrahepatic pathological characteristics similar to BA, but the extrahepatic bile ducts were intact [76]. In another sclerosing cholangitis model, induced by injecting formalin into the common bile duct, inflammatory cells were found around proliferating bile ducts while the hepatocytes remain normal around the portal spaces [77].

The sea lamprey model, on the contrary, does not require injections of any chemicals or special treatment, and the animal progresses through BA spontaneously. The sea lamprey liver can be sampled at any stage of inflammation and fibrosis. However, cirrhosis or liver failure does not occur during developmental BA.

### 3.2. Bile Duct Ligation (BDL) Models

Early experimental BA models include ligation or excision of the common bile duct in adult rats [78]. In the common bile duct excision model, the proximal stump became markedly dilated after excision, leading to death within four weeks after the operation [78]. In contrast, ligation of the common bile duct resulted in recanalization and restitution of the bile duct, and liver function and morphology returned to normal soon afterwards [78].

From the late 1960s to early 1990s, several groups attempted to create an animal model for EHBA but failed to simulate the clinical patterns [79–82], including hyperbilirubinemia, elevated liver enzyme levels, low serum albumin, and high serum hyaluronic acid [83], along with histopathology of bile duct proliferation, cholestasis, giant cell formation, hepatic necrosis, progressive sclerosing cholangiopathy, and various degrees of inflammation [84]. Prenatal ligation of the common bile duct (CBD) in lamb fetuses induced cholestasis and prestenotic dilatation of the CBD, analogous to cystic BA in humans [85, 86]. Ligation of hepatic artery in rabbit or lamb fetuses induced somewhat selective ischemia in IHBDs, jaundice at birth, and hypoplasia or absence of IHBDs in pups [87, 88].

The classical biliary obstruction model is the bile duct ligation (BDL) rat [68, 89, 90]. Cholestatic BDL rats exhibit bile duct proliferation and periportal cell infiltration [66]. Since BA pathology showed cholestatic changes concurrent with bile duct proliferation [91], the BDL model was considered to be representative of human BA pathology [43]. However, the response of young animals to bile duct ligation is not well understood [92]. A selective bile duct ligation model in young animals that simulates the condition of isolated bile duct stenosis commonly observed in a liver-transplanted child after surgery was developed by Tannuri et al., showing alterations in the parenchyma and similar effect in the adjacent nonobstructed parenchyma mediated via paracrine and/or endocrine factors [93].

Due to technical difficulties arising from the size of biliary lobar ducts and the proximity to arterial branches, bile duct ligation was usually performed in adult rats. However, the interlobar accessory bile channels and bile duct collaterals often caused rapid recovery from obstructive cholestasis [94]. Experiments with newborn and young animals were sporadic because of their smaller anatomical structures, the necessity for special anesthetic procedure and microsurgical instruments, and problems keeping the animals alive for the entire experimental period [93, 95]. Nonetheless, significantly different responses were observed between the BDL animals at different ages in bile duct proliferation [96] and fibrogenesis [97]. The increase in desmin expression was more intense and precocious in newborn than in adult rats [64]. Young animals showed slower inflammatory response but a faster regenerative nodule formation and cirrhosis compared to adults [98]. In addition, *α*-fetoprotein was expressed in developing young animals but absent in adults [98]. The development of cirrhosis secondary to biliary obstruction was age-dependent during developmental period and adult animals exhibited more fibrogenesis than young animals [92].

Cholestatic BDL mice also showed considerable protection from ischemic liver injury, measured by the level of transaminase release, histological liver injury, and neutrophil infiltration [99]. After selective bile duct ligation, both the ligated and nonligated lobes showed decreased NF*κ*B, TNF*α* mRNA, and neutrophil infiltration. It appeared that cholestatic liver parenchyma protected the adjacent noncholestatic lobe against ischemic injury [93]. Understanding of the autocrine and paracrine neuromodulators of cholangiocyte proliferation during the progression of cholestatic diseases may help develop therapeutic strategies for BA. Cholangiocyte proliferation is associated with a transdifferentiation of biliary epithelia to express neuroendocrine phenotypes and may provide unique signaling mechanisms for drug development. Preventing or limiting cholangiocyte proliferation and the expression of profibrotic genes and secretion of profibrotic factors during the progression of cholestatic liver diseases could be the first line of defense to control or prevent fibrosis [100].

**Figure 4:**
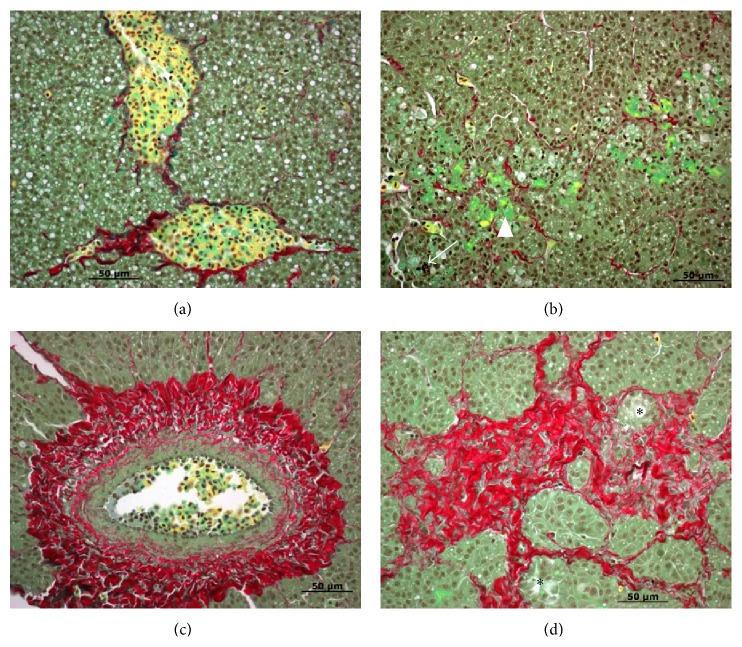
Liver morphology in small parasitic sea lampreys. (a) Steatosis (white vacuoles in hepatocytes) and macrophage recruitment and activation (yellow cells with filopodia). (b) Activated macrophages (yellow cells with filopodia) clearing fibrosis (red), hepatocyte necrosis and bile pigment accumulation (solid white triangle), lymphocyte infiltration (white arrow), and steatosis (white vacuoles in hepatocytes). (c) Collagen deposition around the basement membrane of blood vessels (red) and macrophage recruitment and activation (yellow cells with filopodia). (d) Fibrosis (red) and hepatocyte necrosis (asterisks) and bile pigment accumulation. Paraffin sections were stained with Picro Sirius Red (Gladstone). Scale bar: 50 *μ*m.

**Figure 5:**
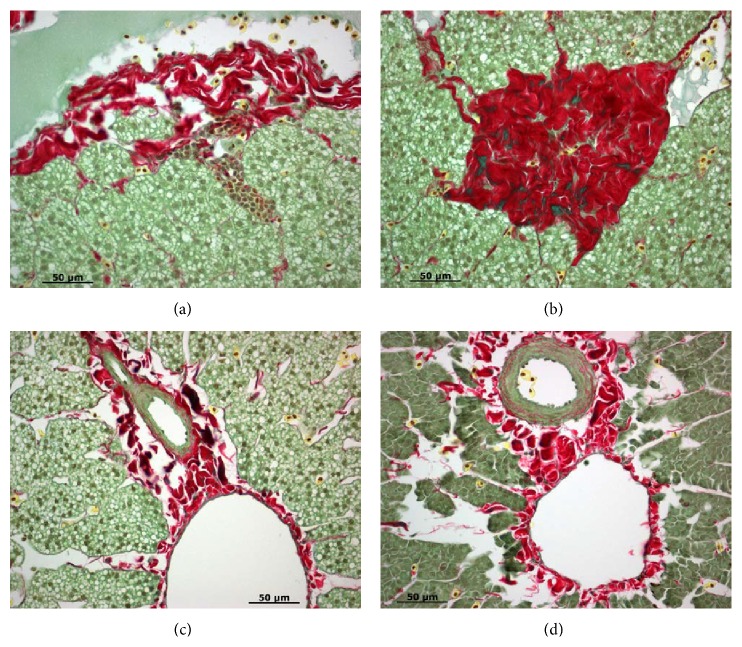
Liver morphology in large parasitic ((a) and (b)), adult male (c) and adult female (d) sea lamprey. (a) Steatosis (white vacuoles in hepatocytes) and macrophage recruitment and activation (yellow cells with filopodia). Note that there are clusters of lymphocytes and macrophages at the fibrotic site. (b) Activated macrophages (yellow cells with filopodia) clearing fibrosis (red) and steatosis (white vacuoles in hepatocytes). (c) Collagen deposition around the basement membrane of blood vessels (red), activated macrophages (yellow cells with filopodia) clearing fibrosis (red) and steatosis (white vacuoles in hepatocytes). (d) Collagen deposition around the basement membrane of blood vessels (red), activated macrophages (yellow cells with filopodia) clearing fibrosis (red) and no steatosis. Paraffin sections were stained with Picro Sirius Red (Gladstone). Scale bar: 50 *μ*m.

Animals with common or lobar bile duct obstruction have been used extensively to mimic human cholestasis. The effects of cholestasis on hepatic morphology, serum and tissue enzymology, metabolic processes, bile secretion, and immunology, as well as more clinically related conditions such as secondary biliary cirrhosis, septicemia, and pancreatic malfunction have been evaluated. However, these animals frequently exhibited short-term cholestasis and the condition could not be sustained. In the sea lamprey model, bile duct obstruction and degeneration are permanent and the processes are similar to human BA (Table 1). Even though cholestasis and fibrosis were transient during metamorphosis (Figures 1–3), the sea lamprey later developed steatohepatitis and fibrosis in parasitic stage and chronic steatohepatitis in adult males (Figures 4 and 5).

### 3.3. Viral Induced Models

A range of hepatotropic viruses can be identified in about 50% of liver biopsies taken during the Kasai procedure [101]. Viral induced cholestasis in mice was first reported by Pickett and Briggs in 1969 [88] and subsequently in the 1980s [79]. They were able to induce inflammation in liver and bile ducts with consequent jaundice but failed to simulate actual atresia of either the intra- or extrahepatic bile ducts. Despite persisting jaundice, early clearance of the virus from the liver, bile ducts, and spleen was observed.

In 1983, Rauschenfels et al. reported a jaundiced rhesus monkey with biliary pathology similar to human BA [101]. This monkey showed persistent high titers of reovirus type 3, supporting Landing's (1974) hypothesis that such hepatotropic viruses could play a role in BA etiology [2]. In 1993, Riepenhoff-Talty et al. observed a temporary biliary obstruction similar to BA after rotavirus (RRV) inoculation in newborn mice [46]. This was the initial step in developing the first viral infected animal model for BA. Intraperitoneal inoculation of Balb/c mice with appropriate titers of RRV within the first 48 h of life leads to an initial viremia which clears later, but the biliary epithelium remains injured [44, 57, 102]. The biliary injury leads to honing of lymphocytes and eventual extrahepatic biliary obstruction, which appears to be mediated by interferon *γ* [103], nuclear factor *κ*B [104], and abnormal activation of the osteopontin inflammatory pathway in the liver [105].

In the RRV EHBA model, the viral infection induces an inflammatory process in the liver and in the intrahepatic and extrahepatic biliary tracts. This pancholangitis leads to irreversible occlusion of the extrahepatic bile ducts, with obstructive cholestasis and intrahepatic bile duct proliferation. The histomorphological changes observed over 3 weeks include edematous swelling with cellular infiltration of the whole biliary tract. In the extrahepatic bile duct, concentric infiltration leads to complete obstruction, sometimes with prestenotic dilatation. Changes in the intrahepatic bile ducts are minimal, and only some parenchymal necrosis is seen [44]. The incidence of RRV-induced EHBA seems to have a critical period, especially within 48 h of birth [57]. As the age of the pups increased, the incidence of cholestasis decreased, and it was impossible to induce EHBA in older mice [106]. Infection of pregnant mice failed to induce EHBA even when the virus was located in the liver [57]. Compared to human BA, nonsyndromatic EHBA has never been observed before birth and after infancy in these mice [57]. The outcome in these mice is fatal and the histopathological findings are similar to EHBA children. However, the extent and distribution of atresia along the extrahepatic bile ducts are variable and do not follow any comprehensible pattern. Therefore, the RRV EHBA model provides no clues for any useful classification of the disease [107]. On the contrary, in lamprey BA, the degeneration patterns in both intra- and extrahepatic bile ducts are predictable and seem to follow a developmental program.

The human lesion is also composed of “bile plugs” within the bile ducts and reactive bile duct proliferation. Bile plugs were not observed in the murine model and bile duct proliferation was mild [108]. Bile duct injury in the RRV-induced murine model was associated with an initial armed CD4^+^ Th1 effector cell releasing IFN*γ* that activated macrophages to produce TNF*α* and nitric oxide. This immune response persisted despite viral clearance and is representative of the hepatic immune profile of human BA at the time of diagnosis [38]. The intrahepatic bile duct infiltrates in human BA are composed of CD4^+^ and CD8^+^ T cells and macrophages with portal tract cellular production of IL-2, IFN*γ*, and TNF*α*. These results validate the use of the RRV-induced murine model as a useful tool to study the pathogenesis of human BA [108]. However, there are some practical disadvantages with RRV-induced murine BA model. Timing and dosing of virus application affect the reproducibility of BA. Injection-related injury to abdominal organs is usually fatal. Cannibalization of pups impedes the harvest of valuable specimens [17]. Occurrence of BA is associated with early postnatal infection but is inversely related to the infective viral dosage. Prenatal infection does not induce jaundice but prevents the offspring from developing cholestasis after postnatal RRV infection [57, 109]. This protection is transmitted transplacentally and not through breast milk [57]. Balb/c mice are the most susceptible strain. The highest incidence of cholestasis (86%) was achieved by infection with 10^6^ PFU/mL RRV within the first 12 h postpartum, resulting in EHBA with a lethality of 100%. However, the later the postpartum infection is, the less effectively it induced EHBA [57]. Versatility of the RRV model is somewhat limited, as sequential investigations cannot be performed in the same animal. Diseased pups are extremely unstable and too small for repeated biopsies or blood sampling. To overcome this, groups of mice have to be sacrificed and each immune cascade has to be simulated in cell lines [110, 111].

For RRV mice to simulate human BA, the cholestatic pups must survive longer than 21 days. So far, no existing animal model allows simulation of this disease in all its clinical diversity [17]. The survival rate of RRV-induced BA mice is only 10%. Approximately 80% of the survivors develop jaundice by the 7th day, stop gaining weight, and die by 21 days after RRV inoculation [44, 102]. A fundamental dissimilarity between murine and human BA is that mice do not develop severe liver fibrosis and portal hypertension. Lethality is 100%, but the causes of death are still unclear because these mice no longer contain the virus. Hence, one of the main tasks is to improve the survival of diseased pups to simulate the full course of BA in order to monitor individual pathophysiological cascade. Daily dosing with 50 *μ*L/g of 5% dextrose in saline can increase pup survival rates to 35% by day 21, and 50% of survivors recovered from BA [112]. The spontaneous recovery suggests that some mice have an acute infection that does not result in permanent biliary obstruction but rather transient obstruction of the bile duct with no subsequent fibrosis. These are clear limitations of the RRV model [112]. On the contrary, the sea lamprey model undergoes a complete BA with no need of inoculation and close to 100% survival rate for months. Furthermore, no special care is required as they do not feed at this stage.

### 3.4. Zebrafish Model

Zebrafish has been used as a model for developmental defects in IHBDs [113]. This animal model is versatile because its rapid and* ex utero* development of large number of embryos and larvae can facilitate gene screening. The conservation of hepatobiliary developmental processes at the molecular level and anatomical function between zebrafish and mammals are evident [114]. Several biliary diseased models have been established in zebrafish, including Alagille syndrome [115], arthrogryposis-renal dysfunction-cholestasis syndrome [116], intrahepatic BA [117], and choledochal cysts [118]. Although a long way from human conditions, the molecular control of both normal and abnormal bile duct development can be elucidated in this model [17]. The sea lamprey model has similar advantages as the zebrafish model. In addition, sea lamprey liver metamorphosis displays the etiology and pathology of BA. However, sea lamprey development is much slower than the zebrafish.

Current animal models are unable to interrupt the progression of liver disease or improve long-term outcome with the native liver [119]. With the same* ex utero* development of large number of larvae, recent annotation of the sea lamprey genome [120], siRNA technique [121], a modified tissue clarification method [122], and advanced liquid chromatography tandem mass spectrometry (LC-MS/MS) bile acid analytical methods [123–126], sea lamprey can provide valuable insights on the molecular mechanisms of BA including the triggering factors for BA, lymphocyte infiltration, fibrogenesis, cholestasis, macrophage activation, and clearance of fibrosis. Utilizing sea lamprey as a BA model will provide much-needed mechanistic insights into the etiology and pathogenesis of BA, specifically the roles of immune system in the initiation and progression of the disease. Furthermore, it can go beyond other animal models to provide a solution for the prevention of BA and clearance of advanced fibrosis.

## 4. Sea Lamprey as a Model for Liver Diseases

Two recent studies showed that sea lamprey evolved unique adaptive and compensatory mechanisms in coping with cholestasis [126, 127], prompting hypotheses on the synthesis, modification, and circulation of bile salts. Understanding these mechanisms can provide information to generate potential adjunct treatments to ameliorate liver damage in patients waiting for or receiving surgical treatments. In a different scope, studies in mature aductular cholestatic and steatohepatitic lamprey can provide crucial information on management of late-stage cholestatic or NASH patients.

### 4.1. Sea Lamprey as a Model for Adjunct Treatment of BA

The lamprey liver undergoes transient cholestasis immediately after the onset of BA [126]. While regulating the rate limiting enzyme* cyp7a1* in bile acid biosynthesis is a key compensatory mechanism in many cholestatic systems [128], cholestasis still results in liver damage even when* cyp7a1* was downregulated. Therefore, downregulation of* cyp7a1* is not the sole answer to alleviate cholestasis because the outcomes are quite different in the sea lamprey [126] and humans [128], suggesting that lamprey may possess other anticholestatic agents in the liver. Such agents, upon elucidation of their mechanisms, could be used in infants waiting for the Kasai operation or liver transplantation and would improve prognosis for these patients if advanced fibrosis can be prevented. The development of an adjunct treatment in addition to surgical treatments is necessary to increase the survival rate of BA patients [129]. Unfortunately, using steroids were not beneficial based on the START trial [129] even though inflammation was thought to be the key factor for BA injuries [130]. Most BA models are induced to simulate the pathological conditions without discriminating secondary or primary causes [17]. Therefore, it is difficult to develop useful adjunct treatments. The sea lamprey can provide answers to such treatments because the liver undergoes transient cholestasis and recovers from it during developmental BA [126].

**Figure 6:**
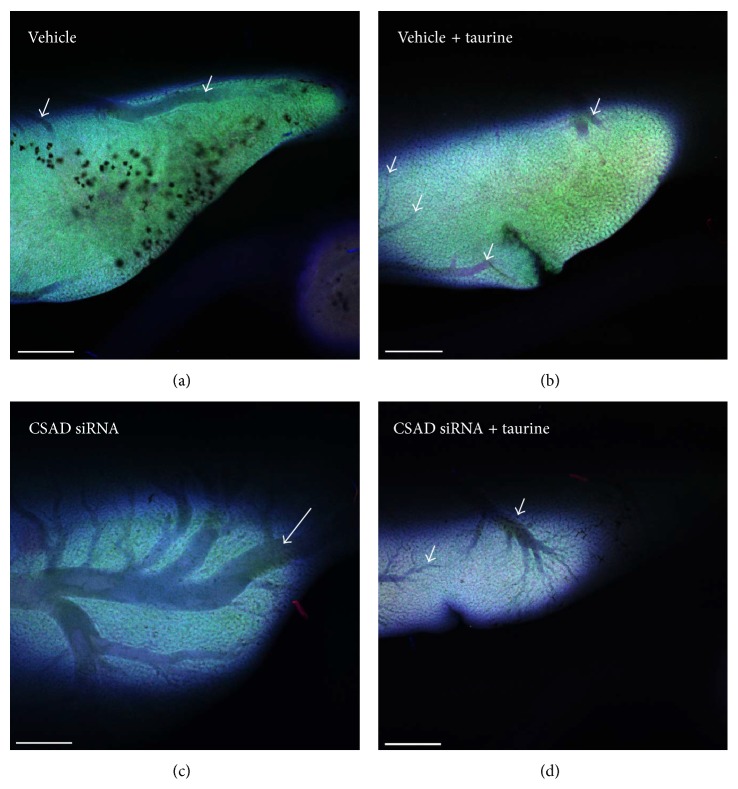
Taurine reduced intrahepatic bile duct dilatation. Larval sea lampreys were injected with vehicle (lipofectamine) and cysteine sulfinic acid decarboxylase (CSAD) siRNA or in combination with taurine. Vehicle-treated liver contained many degenerated cells. Intrahepatic bile ducts were slightly dilated (short white arrows). Treatment with CSAD siRNA induced dilatation of the intrahepatic bile ducts (long white arrow). Taurine seemed to reduce the dilatation of intrahepatic bile ducts. Blue immunofluorescence staining: caspase 3 (apoptosis marker). Green immunofluorescence staining: BCL2 (apoptosis protection marker). Red immunofluorescence staining: CSAD (the rate limiting enzyme for taurine biosynthesis). Whole livers were processed with a modified CLARITY method [122]. Scale bar: 500 *μ*m.

**Figure 7:**
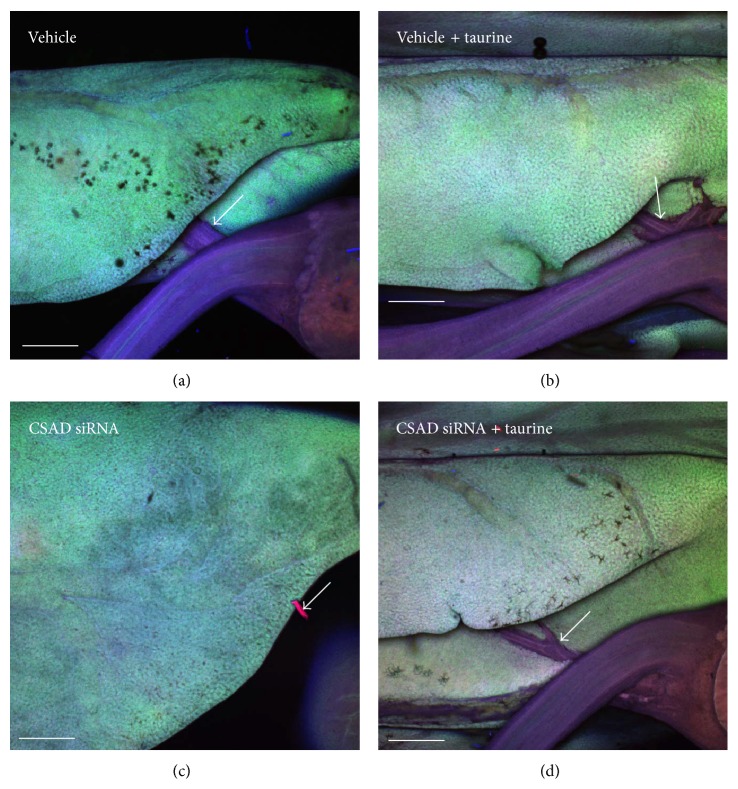
Taurine reduced the degeneration of extrahepatic bile duct. 3D reconstructed confocal images of liver during developmental biliary atresia in sea lamprey. Cysteine sulfinic acid decarboxylase (CSAD) siRNA treatment facilitated degeneration of the extrahepatic bile duct (white arrows). Blue immunofluorescence staining: caspase 3 (apoptosis marker). Green immunofluorescence staining: BCL2 (apoptosis protection marker). Red immunofluorescence staining: CSAD (the rate limiting enzyme for taurine biosynthesis). Whole livers were processed with a modified CLARITY method [122]. Scale bar: 500 *μ*m.

**Figure 8:**
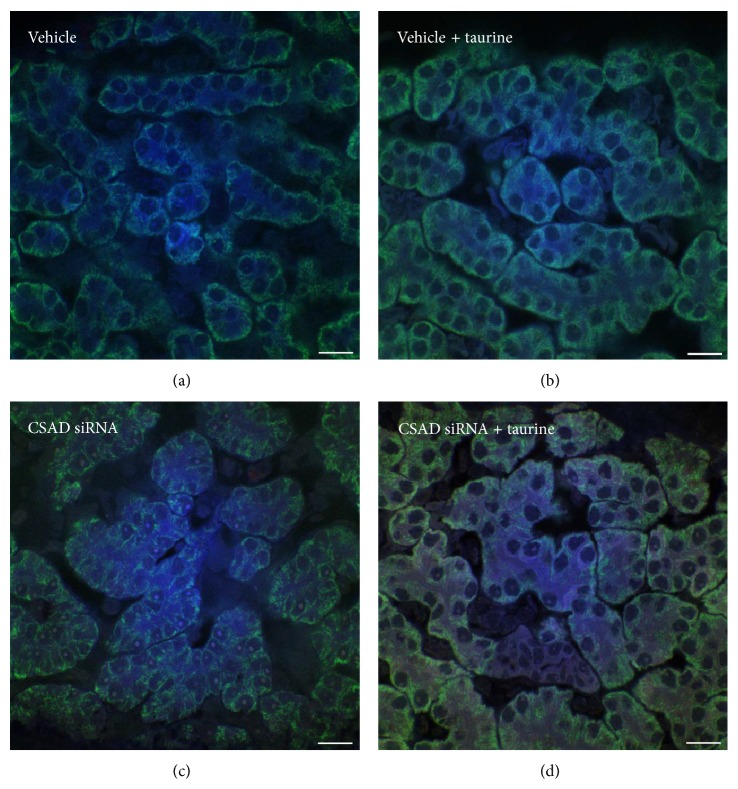
Protective effect of taurine during developmental biliary atresia in sea lamprey. Hepatocytes in the cysteine sulfinic acid decarboxylase (CSAD) siRNA treated group showed pathological cellular morphology. Note that caspase 3 immunofluorescent staining is concentrated at canaliculi between the hepatocytes. Taurine treated hepatocytes showed better membrane integrity and more BCL2 immunofluorescence at the cytoplasm. Blue immunofluorescence staining: caspase 3 (apoptosis marker). Green immunofluorescence staining: BCL2 (apoptosis protection marker). Red immunofluorescence staining: CSAD (the rate limiting enzyme for taurine biosynthesis). Whole livers were processed with a modified CLARITY method [122]. Scale bar: 20 *μ*m.

Taurine (2-aminoethanesulfonic acid) is known to protect against hepatocyte injury induced by hydrazine or CCl_4_. Taurine concentration is higher in sea lamprey liver during developmental BA [126]. Using siRNA to knock down the gene expression of the taurine biosynthetic rate limiting enzyme cysteine sulfinic acid decarboxylase (CSAD) during developmental BA induced dilatation of the IHBDs (Figure 5) and facilitated the degeneration of EHBDs (Figure 6). The hepatocytes showed pathological cellular morphology and the canaliculi showed increased apoptotic marker caspase 3 immunofluorescence (Figure 7). It is interesting that CSAD immunofluorescence was especially concentrated at EHBDs (Figure 6) and addition of taurine seemed to rescue the cellular integrity of the hepatocytes during developmental BA, as hepatocytes showed more normal cellular morphology and apoptosis suppressing factor BCL2 immunofluorescence (Figure 8). This is consistent with the report that taurine exerted cytoprotective effects in isolated hepatocyte from membrane and oxidative damages [131–134].

Although BA accounts for half of neonatal cholestatic patients, other diseases such as infections, Alagille syndrome, and idiopathic causes make up the other half of cases [135]. Understanding the mechanism underlying transient cholestasis in lamprey BA can provide new insights on management of other neonatal cholestatic diseases. The ability of the sea lamprey to survive and thrive after developing BA and subsequent steatohepatitis implies that unique hepatic and extrahepatic adaptive responses have evolved as alternative pathways to minimize metabolic syndromes and bile acid toxicity. Studies in BA patients have documented compensatory changes in bile acid metabolism and transporters that are generally similar to those observed following bile duct ligation in rodents and other chronic cholestatic disorders [128]. Yeh et al. demonstrated that sea lamprey has unique adaptive mechanisms in addition to downregulation of bile acid synthetic enzyme* cyp7a1* [126], of which the transcript level is reduced at both early- and late-stage cholestasis in many animal models and human patients [128]. In addition, the lamprey intestine synthesizes and excretes bile salts into the intestinal lumen during the aductular blood-feeding life stage [126].

### 4.2. Sea Lamprey as a Model for Treatment of Late-Stage Cholestasis

An area of interest concerns the adaptive mechanisms during severe cholestasis when adult sea lampreys reach the final maturation before they spawn and die. It has been shown that bile salt composition shifts dramatically when lampreys transform from juveniles to mature adults [126, 127]. Altered bile salt composition is well described in cholestatic rodents [136] and increases in sulfated bile salts are observed in the serum and urine of cholestatic patients [137, 138]. Understanding the mechanisms involved in these shifts may impact the management of late-stage cholestatic patients. Specifically, the less toxic keto-form of petromyzonol sulfate may be another adaptive mechanism along with renal excretion of bile salts [127]. Whether the conversion of non-keto (3-hydroxy) to 3-keto bile salts contributes to ligand-receptor specificity of pheromones [139], lower toxicities, or both requires further investigation. Nevertheless, the sea lamprey liver does not develop fibronecrosis even at advanced stages of cholestasis [127]. There could be other protective agents such as antioxidants [140] that have yet to be found in this system. During its reproductive stage, the lamprey is a model in which to study late-stage cholestasis, and bile salt composition, and circulation [127, 141].

Two recent studies have shown that the sea lamprey has unique adaptive mechanisms in surviving BA and cholestasis [126, 127]. Lamprey intestine was shown to excrete taurocholate in both* in vivo* and* ex vivo* settings in post-BA animals during their vigorous feeding stage [126]. Bile composition was shown to shift from sulfated bile salts to taurine-conjugated ones immediately after BA and from taurine-conjugated bile salts back to sulfated ones when lamprey reach sexual maturity [126] and release them as sex pheromones [139]. It is not clear why bile salt composition shifts twice in sea lamprey life cycle. Enterohepatic circulation in lamprey is different from that in humans [126], and the lamprey bile salt transporter ASBT does not have affinity for taurocholate [142]. Taurine conjugation may be an adaptation to lamprey BA and subsequent chronic steatohepatitis in parasitic and adult male sea lamprey. Kidneys have also been shown to increase bile salt excretion in severe cholestatic mature lamprey [127]. Understanding the mechanisms underlying bile salt modifications and circulation may provide information for treatments of cholestatic or NASH patients.

Adult lamprey contains normal levels of bilirubin and biliverdin in the plasma but with elevated levels in the liver [143, 144]. Hepatic bile salts in adult lamprey reached millimolar levels, similar to patients with advanced cholestasis or BDL rodents [127]. However, plasma bile salt level in adult lamprey is only ~10 *μ*M, within the normal range observed in mammals. Bilirubin and biliverdin are both antioxidants [145, 146]. Therefore, elevated bilirubin and biliverdin may protect lamprey liver during cholestasis. In addition, adult lamprey converts toxic C24 to C27 bile salts in the liver, hence maintaining normal levels of bile salts and bile pigments in plasma [127]. Adult lamprey seem to cope with BA by reducing bile salt biosynthesis via downregulation of* cyp7a1* and increasing canalicular export through the bile salt export pump Bsep. Renal excretion of bile salts and organic anions in the plasma appears to be facilitated by a strong kidney-specific induction of bile salt transporters Ost*α*, Mdr1, and Bsep [127]. Bile salts and other organic solutes are excreted primarily by way of the kidney in adult lamprey, while only minor amounts are eliminated through intestine [127]. In BDL rodents and cholestatic patients, elevated levels of bilirubin, and bile salts are detected in the urine [137, 147, 148]. Strategies to enhance renal excretion of bile salts and other toxins might be an effective therapy for patients with BA and other forms of cholestasis [127].

To summarize, existing animal models have advanced our knowledge of BA pathogenesis but are unable to provide the etiology. The sea lamprey model can provide pivotal information about BA etiology to prevent bile duct obstruction and fibrosis and adaptive mechanisms to endure NASH and eliminate cholestasis. The abundance of larval and adult lamprey in the Great Lakes region renders such studies feasible. Based on the progression of developmental BA, unique adaptive mechanisms during NASH and cholestasis, and newly established analytical and genomic tools, the sea lamprey can be a valuable model in liver disease research.

## Figures and Tables

**Table 1:** Comparisons between human and sea lamprey biliary atresia.

	Human	Sea lamprey
Bile formation	Yes	Yes
Cholestasis	Yes	Yes
Inflammation	Yes	Yes
Fibrosis	Yes	Yes
Cirrhosis	Yes	No
Liver failure	Yes	No
Downregulation of *cyp7a1* transcripts	Yes	Yes
Extrahepatic excretion sites for bile salts	Kidneys	Intestine, Kidneys, and Gills
